# The Temporal Propagation of Intrinsic Brain Activity Associate With the Occurrence of PTSD

**DOI:** 10.3389/fpsyt.2018.00218

**Published:** 2018-05-25

**Authors:** Yifei Weng, Rongfeng Qi, Feng Chen, Jun Ke, Qiang Xu, Yuan Zhong, Lida Chen, Jianjun Li, Zhiqiang Zhang, Li Zhang, Guangming Lu

**Affiliations:** ^1^Department of Medical Imaging, Jinling Hospital, Medical School of Nanjing University, Nanjing, China; ^2^Department of Radiology, People's Hospital of Hainan Province, Haikou, China; ^3^Department of Radiology, The First Affiliated Hospital of Suzhou University, Suzhou, China; ^4^Key Laboratory of Psychiatry and Mental Health of Hunan Province, Mental Health Institute, Second Xiangya Hospital, National Technology Institute of Psychiatry, Central South University, Changsha, China

**Keywords:** posttraumatic stress disorder, resting-state fMRI, lag mapping, functional connectivity strength, dynamics

## Abstract

The abnormal brain activity is a pivotal condition for the occurrence of posttraumatic stress disorder. However, the dynamic time features of intrinsic brain activities still remain unclearly in PTSD patients. Our study aims to perform the resting-state lag analysis (RS-LA) method to explore potential propagated patterns of intrinsic brain activities in PTSD patients. We recruited 27 drug-naive patients with PTSD, 33 trauma-exposed controls (TEC), and 30 demographically matched healthy controls (HC) in the final data statistics. Both RS-LA and conventional voxel-wise functional connectivity strength (FCS) methods were employed on the same dataset. Then, Spearman correlation analysis was conducted on time latency values of those abnormal brain regions with the clinical assessments. Compared with HC group, the time latency patterns of PTSD patients significantly shifted toward later in posterior cingulate cortex/precuneus, middle prefrontal cortex, right angular, and left pre- and post-central cortex. The TEC group tended to have similar time latency in right angular. Additionally, significant time latency in right STG was found in PTSD group relative to TEC group. Spearman correlation analysis revealed that the time latency value of mPFC negatively correlated to the PTSD checklist-civilian version scores (PCL_C) in PTSD group (*r* = −0.578, *P* < 0.05). Furthermore, group differences map of FCS exhibited parts of overlapping areas with that of RS-LA, however, less specificity in detecting PTSD patients. In conclusion, apparent alterations of time latency were observed in DMN and primary sensorimotor areas of PTSD patients. These findings provide us with new evidence to explain the neural pathophysiology contributing to PTSD.

## Introduction

Posttraumatic stress disorder (PTSD) is one of the most prevalent psychiatric disorders after suffering from severe traumatic events. The major recurrent symptoms including intrusion, avoidance, and hyper-vigilance may seriously impair the ability to get involved in social activities (1). It has been commonly recognized that the disorganized functional neural system played a crucial role in the impairments of fear learning, threat detection, executive function, and emotional modulation in PTSD patients (2). The human brain is a highly ordered dynamic system with ongoing neural activity and rapidly altered neural interaction. Each brain regions or networks serving different functions tend to be active in turn rather than at the same time (3). In the existent literature, the effects of temporal propagation patterns in PTSD are rarely discussed (4). Accordingly, our further research considering propagation patterns of brain activities would be very instructive for thoroughly understanding the neural substrates of PTSD.

The resting-state functional MRI (rs-fMRI) is a straightforward and efficient technique to measure the brain activities. There was adequate evidence across multi-model rs-fMRI studies demonstrating abnormal brain activity in PTSD patients. The variable results have shown the abnormalities in regional areas (e.g., amygdala, insula, and ventromedial prefrontal cortex) (5, 6), inter-networks (6), and whole-brain topology properties (7, 8). Such conventional approaches, which are based on the assumption that functionally connected brain regions were of temporal synchronization, however, might have overlooked the dynamic information (9, 10). Recently, surveys such as that conducted by Garg (11–13) have observed the intrinsic spatiotemporally structured brain activity in mouse and human. Further researches also pointed out that the rs-fMRI data of human could be separated into several temporal function modes with organized reciprocal propagation patterns (14, 15). With the better understanding of spontaneous brain activities, the methods detecting resting-state functional brain dynamics start to draw the attention of neuroimaging scientists; especially, the clinical values of determining temporal latency changes has been highlighted in several diseases, such as localizing the lesions of stroke (16) and epilepsy patients (17).

The emerging data-driven resting-state lag analysis (RS-LA) method, developed by Mitra et al. (18, 19), could provide us with an efficient technique for analyzing the temporal latency at voxel-wise level. This computational approach has been widely used to delineate the temporal dimension of brain communication in both physiological and pathological conditions, including autism (20), epilepsy (21), asleep or awake adults and infants (22, 23). According to their previous reports, the changes in blood oxygen level dependent (BOLD) signal propagation could reflect neural activities, and the propagation structures are sensitive to reveal physiological changes and neural processes in spontaneous activity (15).

A healthy neural system is expected to accommodate the changes of internal or external conditions in real-time, wherefore shows the greater temporal variability compared with unhealthy one (4). Recent dynamic functional connectivity studies have unveiled the temporal variation reduction in PTSD patients (4, 24). We hypothesized that disordered communication between vulnerable brain regions or networks might contribute to the abnormalities in PTSD patients. Accordingly, it is considerate to take into account the dynamical changes of functional connectivity when investigating the underlying neural pathophysiology of PTSD.

In this study, we introduced RS-LA method to explore the temporal propagation patterns of brain activity in PTSD patients. The conventional rs-fMRI analysis was also applied to the same datasets so that it could provide comparison and supplement to the novel technique. A comprehensive understanding of the neural dynamics would make a difference in revealing the exact neuropathological mechanism of PTSD.

## Methods

### Subjects

In June 2014, Typhoon Rammasun struck Hainan Province and caused at least 14 deaths. More than a thousand people were trapped and almost drown in the worst hit area. From November 2014 to January 2015, we ultimately recruited 70 trauma-exposed survivors. This study was conducted according to the declaration of Helsinki and was granted permission by the ethics committee of Jinling Hospital, People's Hospital of Hainan Province and the Second Xiangya Hospital. Before the examination, all the participants did not undergo any anti-depressant or psychotherapy. The inclusion criteria of the current study were as followed: age 18–65 years; right-handedness; no use of psychiatric medication or substances abuse; without MR imaging contraindications. Subjects with any history of head trauma, loss of consciousness, long-term significant physical conditions, neurologic or psychiatric disorders except for depression and anxiety should be excluded. After considering the rigid requirements, we totally excluded 10 trauma-exposed subjects for failing to obtain complete imaging data (*n* = 3), excessive head movement (*n* = 3), brain infarction (*n* = 1), denture-artifacts (*n* = 2), and pregnancy (*n* = 1). Thirty demographically matched healthy controls (HC) without trauma-exposure were also enrolled in our study. Every participant provided written informed consent prior to MRI scan and neuropsychological assessments.

### Psychometric assessments

All the typhoon survivors were screened with PTSD checklist-civilian version (PCL_C) (25), a 17-item self-report instrument designed to assess symptoms of PTSD (26). Those subjects who PCL_C scored more than 35 were further administrated with Clinician-Administrated PTSD Scale (CAPS) to estimate the frequency and intensity of each sub-symptom including re-experience, avoidance, and hyper-vigilance (27). The remaining subjects scoring <30 were considered as the trauma-exposed controls (TECs). The comorbidities with other psychiatric disorders were confirmed via the Structured Clinical Interview for DSM-IV (28). Additionally, emotional assessments including Self-Rating Anxiety Scale (SAS) (29) and Self-Rating Depression Scale (SDS) (30) were conducted on all participants to estimate emotional status.

### Data acquisition

The resting-state functional MR imaging was acquired with a 3.0 Tesla MR scanner (Skyra, Siemens Medical Solutions, Erlangen, Germany) equipped with the standard head coil. Each subject was instructed to lay supine, rest, and keep his or her eyes closed with the head still during MRI scanning. Firstly, the routine diagnostic T1 weighted image and T2 fluid-attenuated inversion-recovery image acquisitions were conducted to rule out subjects with structural brain lesions. The resting-state fMRI data were then acquired using a single-shot, gradient-recalled echo planar imaging (250 volumes, repetition time [TR]/echo time [TE] = 2,000/30 ms, flip angle = 90°, image matrix = 64 × 64, FOV = 230 × 230 mm^2^, slice thickness = 3.6 mm, 35 axial slices with no intersection gap). Each volume was whole-brain coverage and aligning along the anterior-posterior commissure. Additionally, high-resolution T1-weighted 3-Dimension anatomical images were obtained in the sagittal orientation using a rapid gradient-echo sequence (TR/TE = 2000/1.97 ms, flip angle = 9°, image matrix = 256 × 256, FOV = 256 × 256 mm^2^, slice thickness = 1 mm, 176 slices).

### Resting-state fMRI data preprocessed

Initial data preprocessing was conducted using the Data Processing Assistant for rs-fMRI advanced edition (DPARSFA, http://www.restfmri.net) (31) based on MATLAB (The Math Works, Inc., Natick, MA, USA) platform. The first 10 volumes of each fMRI data were removed for the signal equilibrium. Then, the subsequent procedures were performed on the remaining 240 volumes, including slice timing, realignment, and co-registered with the anatomical scan. The co-registered data was further segmented into gray matter, white matter (WM), and cerebrospinal fluid (CSF) and normalized into standard Montreal Neurological Institute (MNI) space with a final voxel size of 3 × 3 × 3 mm^3^. Additionally, preprocessing for temporal lagged analysis included spatial smoothing by convolution with an isotropic Gaussian kernel of 8 mm, removal of linear trends to correct for general signal drift and band-pass filtering (0.01–0.08 Hz) to reduce low-frequency noise (32, 33). The final set of nuisance covariates including the six head motion parameters, average signals from WM and CSF, and the time series averaged over the brain (34) were regressed. Moreover, we applied frame censoring to each group by using the DVARS (differentiated rms variance) measure (35) with a threshold of 0.5 % root mean square frame-to-frame intensity change (36). Therefore, the criteria removed 8.10 ± 2.79, 9.93 ± 3.16, and 4.36 ± 1.07% of frames per individual, respectively, in PTSD, TEC, and HC group. Subjects with < 195 frames should be excluded. There were no statistically significant differences in the amount of censored time points between groups. Lastly, de-noising was consequently conducted to improve “cosmetic” using a combination of strategies similar to previous studies (37–40).

### Computation of lag between BOLD time series

Our method for computing lags between time series was according to previously published literature (19). Considering the temporal features of intrinsic brain activity in its latency structure (Supplementary Figure 1), we evaluated the lagged cross-covariance functions according to the following formula (19):

(1) $$\begin{array}{l} Cxixj\left(\tau \right)=\;\frac{1}{T}\int xi\left(t+\tau \right)\times xj\left(t\right)dt\; \end{array}$$

where τ represent the time lag. The value of τ where *Cxixj* (τ) exhibited an extremum defines the temporal lag between signals *xi* and *xj*. T is the interval of integration. BOLD time series are aperiodic (41). Accordingly, it would almost always generate a single, well-defined extremum when calculated by the cross-covariance functions, typically in the range ±1 s.

Voxels were defined by dividing the gray matter mask in atlas space into 6-mm isotropic cubic regions. Given the time series {*x*1(*t*), *x*2(*t*), …, *xn*(*t*)}, extracted from all voxels (*n* = 5,797 in current study), finding all τ*i, j* corresponding to the extrema of *Cxixj* (τ) yields the antisymmetric, time-delay (TD) matrix:

(2) $$TD=\left[\begin{array}{ccc} \tau_{1,1} & \cdots & \tau_{1,n}\\ \vdots & \ddots & \vdots \\ -\tau_{1,n}\; & \cdots & \tau_{n,n} \end{array}\right]$$

Then, group level latency projections were obtained by calculating the projections that computed as the mean across the columns of TD matrix at subject level and then averaging. All the results were represented in three-dimensional image formats using the BrainNet Viewer (http://www.nitrc.org/projects/bnv/).

Besides, we evaluate the voxel-wise whole-brain functional connectivity strength (FCS) to offer a reference for comparison with the results of RS-LA. Conventional FCS was defined by computing the average functional connectivity between a given voxel and all other voxels in the brain (42). The procedures of data preprocessing were almost the same as RS-LA, except that data smoothing was implemented after the Pearson correlation coefficients were converted using Fisher r-to-z transformation. All the processes were calculated by using DPARSFA. Voxels with high FCS (> mean) were identified as functional hubs, which indicated that they were highly connected to the rest of the brain.

### Statistical analysis

SPSS 22.0 (SPSS INC, Chicago, IL, USA) was used to analyze the demographic and clinical data. The Chi-square test was applied to evaluate gender difference among three groups. Normally distributed material expressed as mean ± standard deviation was assessed by one-way analysis variance (ANOVA) and the homogeneity of variance in these data was examined by the Bartlett test. When the ANOVA analysis revealed significant differences, *post-hoc* analysis was employed for inter-group comparisons.

Due to the group differences in education level, RS-LA and FCS differences among the three groups were analyzed by using ANOVA with educational level as covariates, followed by *post-hoc t*-test to confirm the between-group differences. Regardless of the trauma effect, we also compared the group differences between PTSD patients and all control subjects with two-sample t test. The above analyses were respectively based on the Statistical non-Parametric Mapping software for RS-LA (SnPM13, http://warwick.ac.uk/snpm) and Statistical Parametric Mapping software for FCS (SPM8, http://www.fil.ion.ucl.ac.uk/spm/). All the maps were multiple compared and corrected with AlphaSim Program, with the threshold set at *P* < 0.01 and the voxel numbers of the cluster larger than 75, which corresponded to *P* < 0.05.

Finally, to investigate the association between latency structures and PTSD symptoms, the average latency values extracted from the clusters with significant lagged differences were correlated with the clinical measurements. Spearman correlation analysis with the significant level of *P* < 0.05 (two-tail test, Bonferroni corrected).

## Result

### Demographical and clinical characteristics

Ultimately, 27 drug-naïve patients with PTSD (48.41 ± 10.32 years; 7 males, 20 females), 33 TEC (48.45 ± 7.48 years, 7 males, 26 females), and 30 HC (49.87 ± 6.11 years, 7 males, 23 females) underwent data analysis. The detailed demographics and clinical characteristics are shown in Table 1. There were no group differences for age and gender distribution. However, the education level of HC group was higher than TEC and HC group. Besides, there were also significant differences of SAS and SDS among the three groups, which the scores in PTSD and TEC groups are much higher than HC group, and the scores of PTSD were also higher than TEC group.

**Table 1:** Summary of demographic and clinical data.

	**PTSD (*n* = 27)**	**TEC (*n* = 33)**	**HC (*n* = 30)**	***P*-value**
M to F ratio	7:20	7:26	7:23	0.912^a^
Education (year)	6.41 ± 3.35	6.97 ± 3.36	9.73 ± 3.29	<0.001^b^
Age (year)	48.41 ± 10.32	48.45 ± 7.48	49.87 ± 6.11	0.729^b^
SAS score	52.63 ± 10.63	33.09 ± 6.60	28.77 ± 4.38	<0.001^b^
SDS score	55.67 ± 10.56	33.06 ± 7.38	26.83 ± 5.72	<0.001^b^
PCL_ C score	53.74 ± 8.46	28.94 ± 5.44		<0.001^c^
CAPS total score	78.18 ± 19.29			
*Intrusion*	24.52 ± 7.27			
*Avoidance*	28.07 ± 8.26			
*Hyper-vigilance*	25.59 ± 6.92			

*Data are means ± standard deviations. PTSD, posttraumatic stress disorder; TEC, traumatic exposed controls; HC, healthy controls; M, male; F, female; PCL, PTSD Checklist-Civilian version; CAPS, Clinical-Administered PTSD Scale; SAS, Self-Rating Anxiety Scale; SDS, Self-Rating Depression Scale*.

a *P-value calculated with Chi-square test*.

b *P-value calculated with one-way analysis of variance*.

c *P-value calculated with two-sample t-test*.

### Resting-state lag analysis

**Figure 1:**
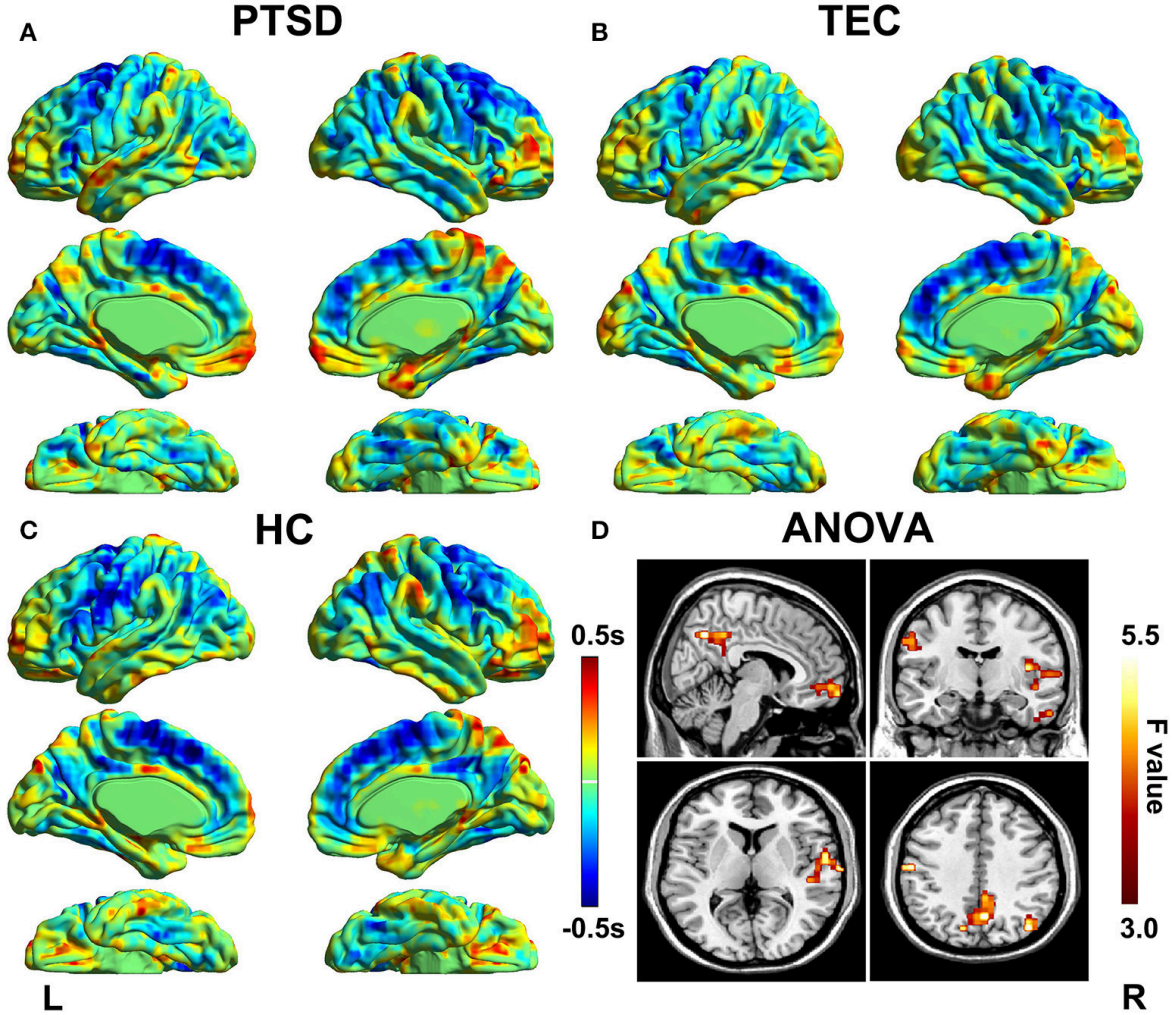
Lag projection maps in PTSD, TEC, and HC group and the ANOVA result. Maps **(A–C)** show the latency results of each group, which represent whether the cluster is on average earlier or later compared with the rest of the brain. The propagation of BOLD signal is measured on a time scale of ±0.5 s. Map **(D)** shows group differences in latency results among the three groups. Color in red indicates statistically significant clusters.

Latency projections results obtained from the PTSD, TEC, and HC group are displayed in Figure 1. All group level latency maps span 1 s between the earliest and latest brain regions. It exhibited that time latency pattern of the early and late brain structures were highly symmetrical and spatially distinct in each group. Additionally, we could found significant group differences among the three groups.

**Figure 2:**
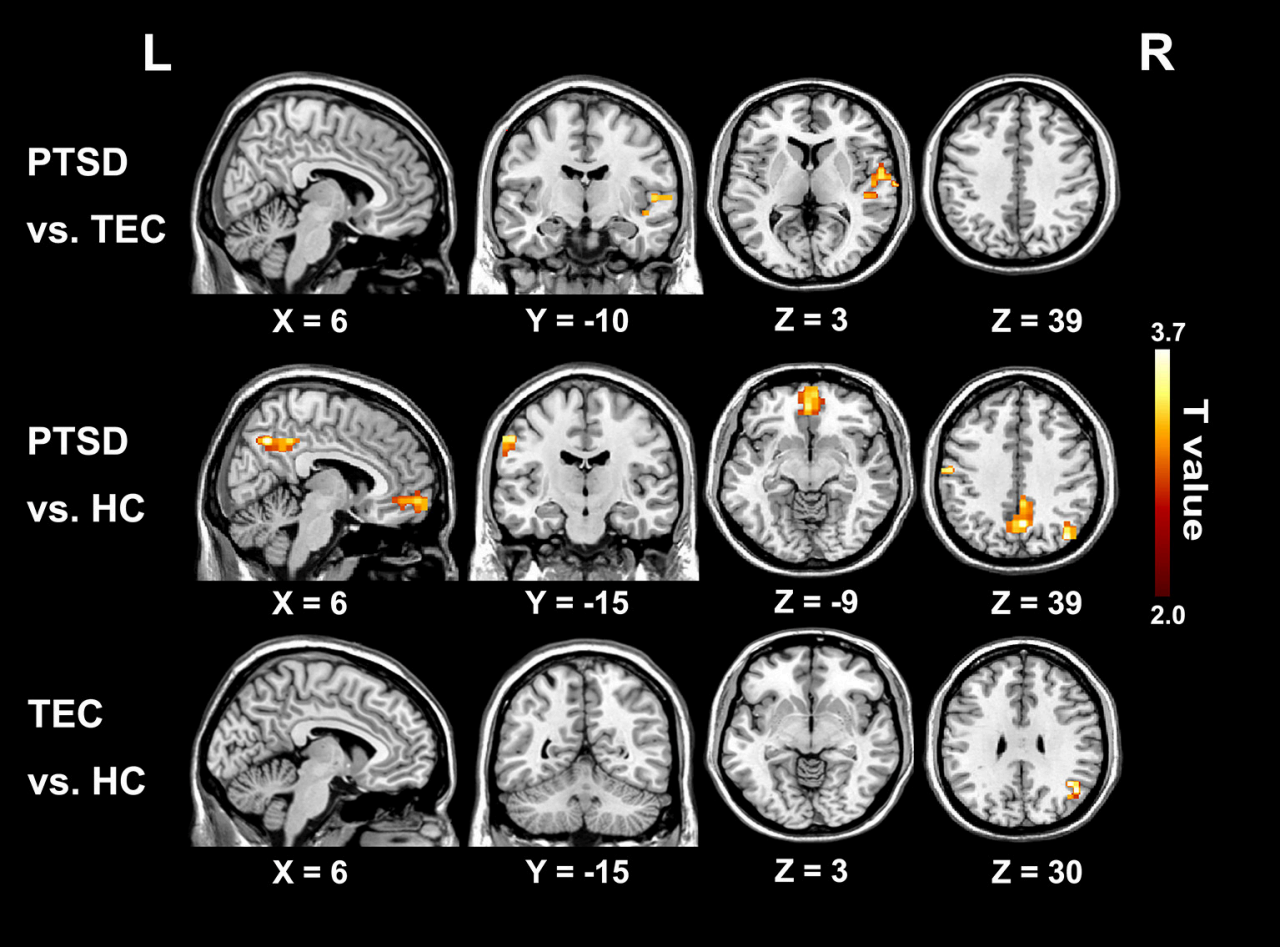
Significant differences in lag structure estimate between groups. Areas with significant group difference of RS-LA are in right superior temporal gyrus of PTSD group when compared to the TEC. When compared to the HC, the differences are in left pre- and post-central cortex, medial prefrontal cortex, posterior cingulate cortex/precuneus, and left angular. Lag structure difference in right angular can also be observed in the TEC group when compared with the HC group. There is no significant difference in brain regions with latency shifting toward earlier among the three groups.

The group differences among three groups are showed in Table 2 and Figure 2, The changes in time latency of PTSD patients were the prominent shift toward later in left superior temporal gyrus (STG), posterior cingulate cortex/precuneus (PCC/PCu), middle prefrontal cortex (mPFC), right angular, and left pre- and post-central cortices (Pre/PostCG). Most of these differences could be found when comparing the PTSD group with HC group, while only the STG was the exception as the result of comparison between PTSD and TEC group. Besides, we observed similar time latency in right angular in TEC group compared to HC group. There was no significant difference in brain regions with time latency shifting toward earlier among the two trauma groups.

**Table 2:** Brain regions show latency differences among the three groups.

**Brain region**	**MNI coordinated (x, y, z)**	**Number of voxels**	**Peak *F*-value**	**Peak *P*-value**
STG_R	51, −33, 9	88	6.43	<0.001
Pre/PostCG_L	−51, −3, 21	79	8.00	<0.001
mPFC	−3, 45, −15	86	6.71	<0.001
PCC/PCu	3, −63, 39	87	6.12	0.003
Angular_R	39, −75, 39	80	7.27	0.030

*MNI, Montreal Neurologic Institute; R, Right; L, Left; STG, superior temporal gyrus; Pre/PostCG, pre- and post-central gyrus; mPFC, medial prefrontal cortex; PCC/PCu, Posterior cingulate cortex; PCu, Precuneus*.

### Correlation result

**Figure 3:**
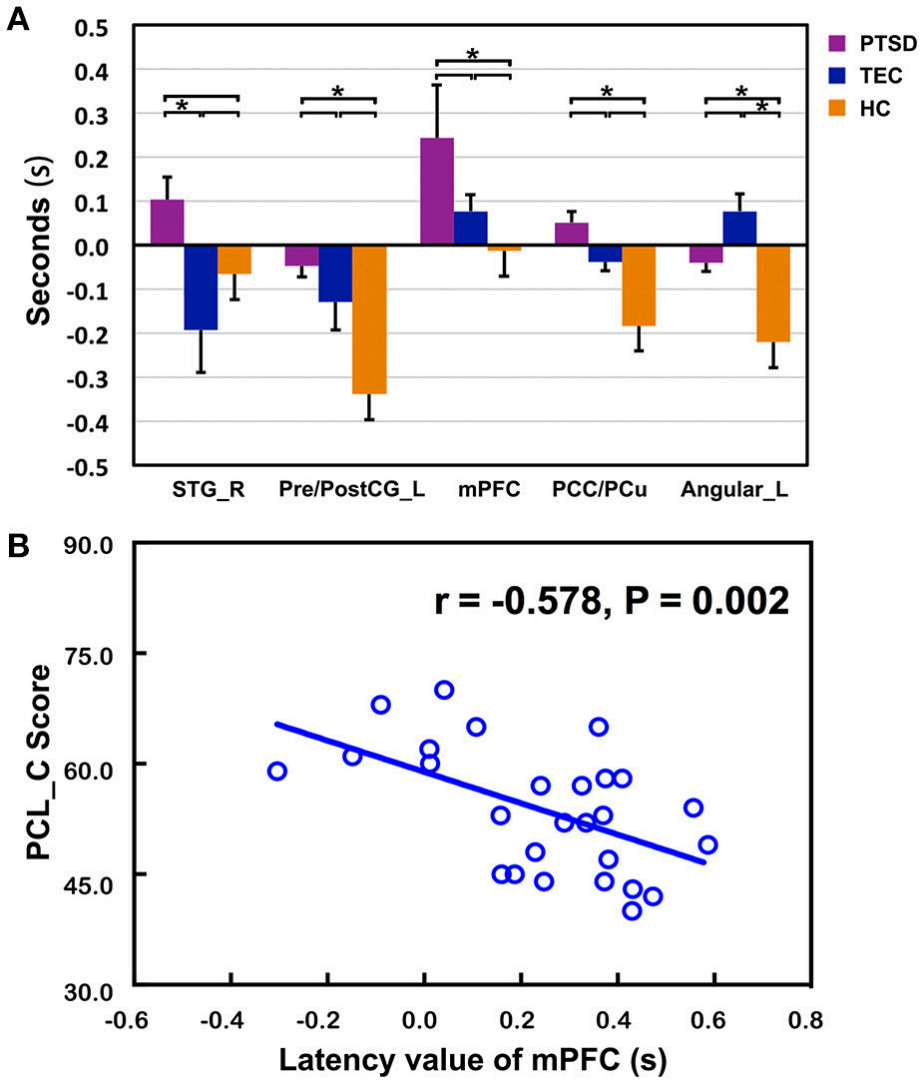
Comparisons of latency values in the region with group differences among the three groups and the significant correlation with clinical assessments. **(A)** Displays detailed latency values of each group in the form of a bar chart. R, Right; L, Left; STG, superior temporal gyrus; Pre/PostCG, pre- and post-central gyrus; mPFC, medial prefrontal cortex; PCC/PCu, Posterior cingulate cortex; PCu, Precuneus; *Significant different (*P* < 0.05); *error bar* standard error of mean (SEM); **(B)** shows the significant correlation of time latency value of mPFC and the PCL_C scores in the PTSD group.

The time latency values with significant group differences were extracted and displayed in the form of bar chart at the group level (Figure 3A). The results of correlations between clinical measurements and RS-LA values of all significant clusters in both PTSD and TEC were reported in the Supplementary Table 1. In PTSD group, our further analysis found negative correlation between the time latency values of mPFC and the PCL_C scores (*r* = −0.578, *P* = 0.002 < 0.05, Figure 3B), which is absence in the TEC group (*r* = 0.030, *P* = 0.868).

### Resting-state function connectivity strength

**Figure 4:**
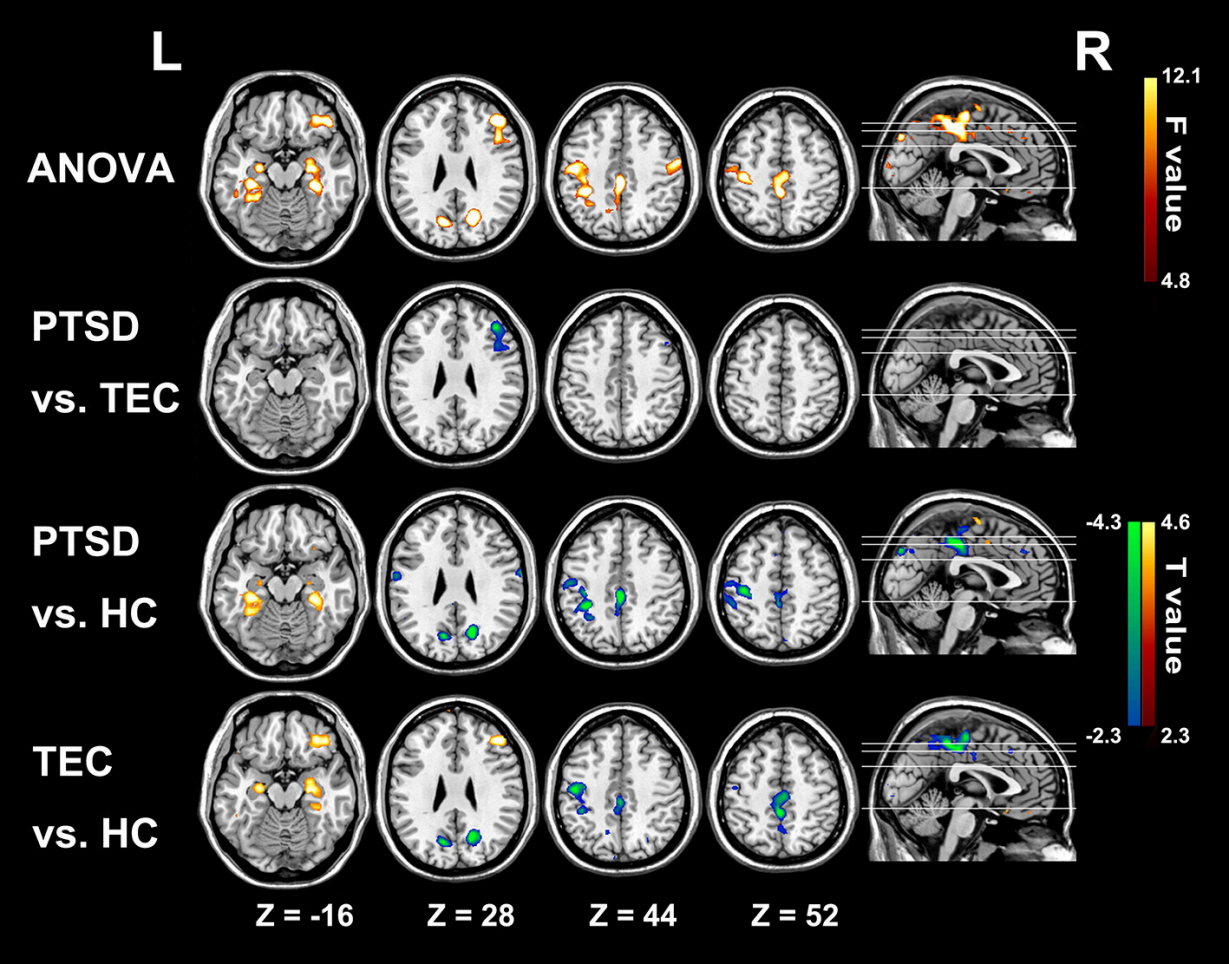
Significant group differences results of functional connectivity strength among the three groups. In the PTSD group, significant increased functional connectivity strength regions mainly distribute in bilateral parahippocampus/hippocampus; and the hypo-activity regions are in right middle and inferior frontal gyrus, PCC/PCu, middle cingulate cortex, bilateral cuneus, and left Pre/PostCG. Most of these alterations can also be observed in the TEC group. Colors in red and blue respectively indicate significant increase and decrease in the *post-hoc* statistical result.

Conventional resting-state functional connectivity strength (RS-FCS) differences of the three groups are shown in Figure 4. In the PTSD group, visual inspection reveals aberrant brain hyper-activity in bilateral parahippocampus/hippocampus, as well as the hypo-activity in right middle and inferior frontal gyrus, PCC/PCu, middle cingulate cortex, bilateral cuneus, and left Pre/PostCG. It should be noted that most of these alterations could also be observed in the TEC group. Additionally, the noticeable brain regions of RS-LA and FCS comparison between PTSD patients and all controls did not show the specialness (Supplementary Figure 2).

## Discussion

In the current study, by using RS-LA, we identified the lagging structures of intrinsic brain activities in PTSD patients. The disturbance of brain activities was not only involved in the inter-regional connectivity but the laggingly driven of the specific brain regions, mainly distributing in DMN and the primary sensorimotor areas. Time latency values of the mPFC negatively correlated with the severity of PTSD symptoms. Moreover, comparing with the conventional functional connectivity method, results of LA-RS appeared to be more complementary to describe the brain activity changes in PTSD patients. These observations are significant in shedding the light of potential relations between the structures with apparent time latency and the occurrence of PTSD.

The present study revealed predominant temporal postponement of mPFC, PCC/PCu and angular gyrus in PTSD group. These regions have been previously identified as major regions of DMN. The explanation for our results relies on the underlying neural physiological basis of lag structure. It has been speculated that the propagation of spontaneous low-frequency activity in lag structure might be concerned with the regional variation in time latency of either neurovascular coupling or transduction of neuronal activities into BOLD signals (19, 43). Numerous evidence has indicated that the DMN is a major locus of intrinsically propagated brain activity, which serves as the hub of neural information transmission and more susceptible under the neural pathological conditions (13, 44). The time latency values shifting toward later could be represented as the slower triggering and propagation of brain activity, as well as the disturbance of ordered brain activities. Accordingly, our observation of time latency differences in DMN is consistent with those pieces of literature that proposed DMN abnormalities in PTSD patients (6). DMN is a vital network responsible for the internal thought and autobiography memory (45, 46). The disorganization in DMN might give rise to severe consequences, such as dissociation symptoms, somatization, emotional disorders and self-perception dysfunction (47, 48). It was supported by our observation of the negative correlation between time latency values of mPFC and PTSD clinical measures.

Additionally, in PTSD group, the significant time latency of right STG was found compared with TEC group and that of the Pre/PostCG was also found relative to HC group. The structural abnormities in these regions have been widely reported, which mainly demonstrated increased gray matter density (49, 50). So far, many mechanisms have attached the importance to the role of brain structure in explaining how brain abnormalities propagation, which might be involved in vulnerability caused by particular co-expression of genes between certain regions, transneuronal spread of misfolded proteins along axonal pathways, and so on (51, 52). Accordingly, the correlation between anatomic changes and the lag structure alterations worth our future investigation. Recent evidence (53) has suggested that the disturbance of functional activities in STG and Pre/PostCG of PTSD patients correlated with the severity of subsequent symptoms at the early stage. The impairment in both two regions could be speculated to be responsible for the disturbance of trauma memory network in charge of its auditory memories combining with motor programs (53).

In RS-FCS result, we equally observed the abnormal activities in PCC/PCu and left Pre/PostCG gyrus in PTSD patients and TEC group. By contrast, our finding of time latency in these two regions was only observed in PTSD group, thus complementarily expressing that the lagged propagation of brain activities might be pertinent to the patients. Jin el al also found that PTSD patients with abnormal static connectivity would couple with altered temporal variability of connections (4). Especially, the aberrant static and dynamic brain activities in similar regions (i.e., Pre/PostCG) were found in the both researches. The similarities possibly help to explain why the temporal lag could only be observed in PTSD patients; nevertheless, more works are still needed to determine whether these intriguing correspondences are general in any way. Unexpectedly, the localization of PCC in FCS method was different from that in RS-LA, and the abnormalities of mPFC were confined to the RS-LA results. Concurrently, altered brain activities of bilateral hippocampus, the middle cingulate cortex, and the right middle and inferior frontal gyrus could be only found by FCS in both PTSD and TEC groups, but failed to be identified by RS-LA method. As for above-mentioned discrepancies, Mitra et al. has suggested that there is no simple relation between lag and static zero-lag temporal correlations (15, 18). Under the logical extreme circumstances, synchronous zero-lag functional connectivity contains no lags, while a system with a single set of lags is not synchronous (18, 54). Moreover, the two distinct time-scale methods can provide us information of functional neural segregation and integration: zero-lag functional connectivity can map separated functional area; and lag threads demonstrate how the distinct functional modules could be integrated over few seconds (55), although the exact physiology served by lag threads still remains unknown. In a word, these results remind us that diverse rather than single method should be taken into consideration when studying the exact neural pathology of PTSD.

## Limitation

To our best knowledge, this is the first study employing RS-LA to investigate the dynamically intrinsic brain activity in PTSD patients. We included the traumatic controls experiencing the same trauma events to reduce the impact of interference factors. However, some limitations need to be acknowledged. Firstly, it should be regarded as a preliminary study because of the relatively small samples. Secondly, there are significant educational level differences among the three groups, and we regressed the educational effect as a covariate to minimize the influences. Thirdly, we only retrieved the clusters with significant group differences in lagged structures to focus on the latency patterns related to PTSD, while further discussion on static results is needed in our future works. Lastly, our exploration of the clinical correlates of altered lag structure is limited to the clinical assessments collected in the current study. More detailed and comprehensive clinical materials are required in the future.

## Conclusion

In summary, we demonstrated that the time latency patterns related to the occurrence of PTSD. The altered propagation of BOLD signals markedly happened in DMN and primary sensorimotor regions. We also observed that the changes in some DMN regions might be associated with PTSD severity. Compared with conventional methods, these results detected by the novel technology provided evidence that aberrant propagation of brain activities would contribute to PTSD. Notably, further researches, especially with longitudinal designs, are still needed to confirm the potential value for clinical application.

## Ethics statement

This study was in accordance with the declaration of Helsinki, and was approved by the ethics committee of Jinling Hospital, People's Hospital of Hainan Province and the Second Xiangya Hospital of Central South University. All participants provided written informed consent after a detailed description of this study.

## Author contributions

YW was involved in the literature review, experimental design, data analysis and writing of the manuscript. RQ, FC, ZZ, and GL designed the study. JK, QX, and YZ analyzed the data. LC, JL, and LZ acquired the neuropsychological data, which all authors reviewed and approved for publication.

### Conflict of interest statement

The authors declare that the research was conducted in the absence of any commercial or financial relationships that could be construed as a potential conflict of interest.

## Supplementary material

Supplementary Figure 1 We select a healthy sample, and then extracted the rs-fMRI signals of two major brain regions related to the results and calculated the cross-correlation coefficient to demonstrate how correlation changed with different time lags. **(A)** The sagittal plane shows the two major areas where we extract the rs-fMRI signals. **(B)** The lagged cross-correlation coefficient changes within a random repetition time. The extremum (yellow maker) shows the lag between corresponding time series and the coefficient. **(C)** Two hundred and forty seconds of sampled time series extracted from the brain regions.

Supplementary Figure 2 We perform the two-sample *t-*test analysis between the PTSD patients and all controls (TECs & HCs). For RS-LA result, the significant latency structure is found in mPFC. As for FCS, the significant increase is observed in bilateral parahippocampus; and the decrease is in right middle and inferior frontal gyrus, PCC/PCu, left cuneus and left Post CG. All the noticeable brain regions of RS-LA and FCS comparison between PTSD patients and all controls are included in the ANOVA results.

Supplementary Table 1 The results of lag values in all clusters with significant group differences correlate with clinical measurements.
