# Supplementary figures and images for: The Temporal Propagation of Intrinsic Brain Activity Associate With the Occurrence of PTSD
Source: Front Psychiatry. 2018 May 25;9:218. doi: 10.3389/fpsyt.2018.00218 (PMC5980985; doi:10.3389/fpsyt.2018.00218)

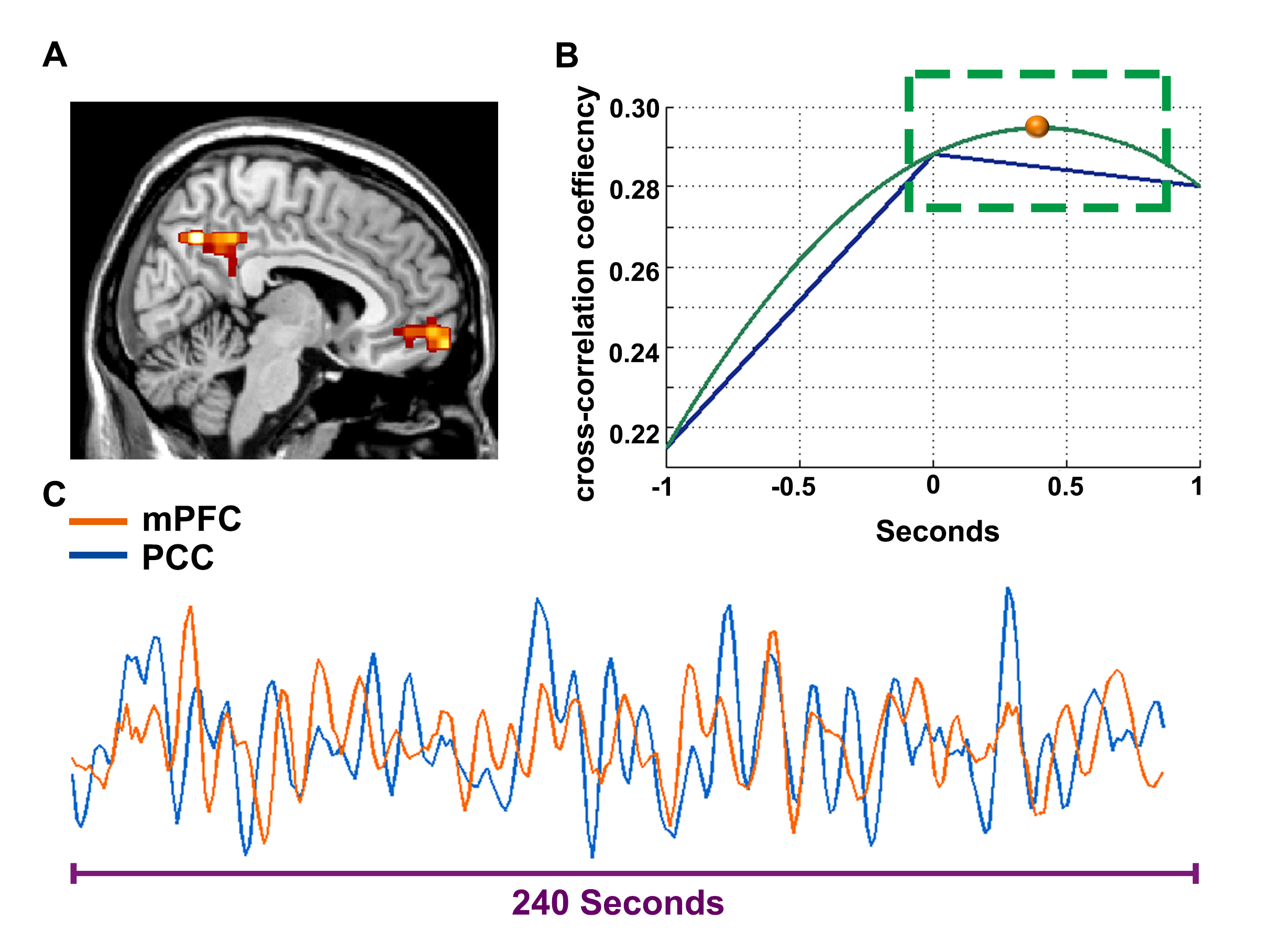

Supplement: Supplementary Figure 1 — We select a healthy sample, and then extracted the rs-fMRI signals of two major brain regions related to the results and calculated the cross-correlation coefficient to demonstrate how correlation changed with different time lags. (A) The sagittal plane shows the two major areas where we extract the rs-fMRI signals. (B) The lagged cross-correlation coefficient changes within a random repetition time. The extremum (yellow maker) shows the lag between corresponding time series and the coefficient. (C) Two hundred and forty seconds of sampled time series extracted from the brain regions. [file Image_1.TIF]

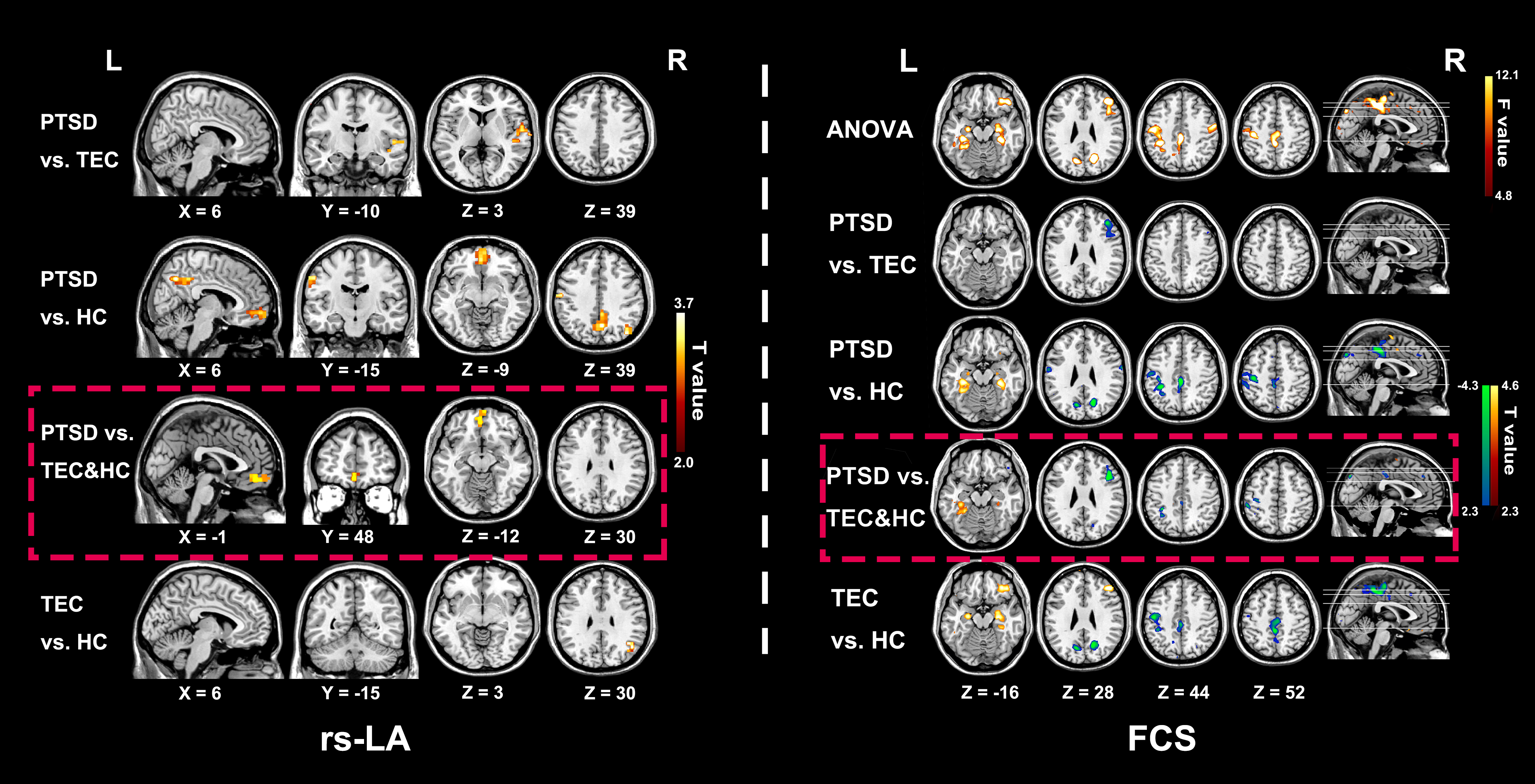

Supplement: Supplementary Figure 2 — We perform the two-sample t-test analysis between the PTSD patients and all controls (TECs & HCs). For RS-LA result, the significant latency structure is found in mPFC. As for FCS, the significant increase is observed in bilateral parahippocampus; and the decrease is in right middle and inferior frontal gyrus, PCC/PCu, left cuneus and left Post CG. All the noticeable brain regions of RS-LA and FCS comparison between PTSD patients and all controls are included in the ANOVA results. [file Image_2.TIF]
