# Supplementary material for: The Temporal Propagation of Intrinsic Brain Activity Associate With the Occurrence of PTSD
Source: Front Psychiatry. 2018 May 25;9:218. doi: 10.3389/fpsyt.2018.00218 (PMC5980985; doi:10.3389/fpsyt.2018.00218)
Supplement: Supplementary Table 1 — The results of lag values in all clusters with significant group differences correlate with clinical measurements. [file Table_1.DOCX]

**Supplementary Table.** The results of lag values in all clusters with significant group differences correlate with clinical measurements.

|  |  | PTSD | | TEC | | HC | |
| --- | --- | --- | --- | --- | --- | --- | --- |
| SAS |  | **r** | **P** | **r** | **P** | **r** | **P** |
|  | STG_R | 0.163 | 0.416 | 0.097 | 0.590 | -0.326 | 0.078 |
|  | Pre/PostCG_L | 0.088 | 0.664 | -0.106 | 0.557 | 0.079 | 0.677 |
|  | mPFC | -0.346 | 0.077 | -0.128 | 0.479 | -0.093 | -0.627 |
|  | PCC/PCu | 0.075 | 0.712 | 0.035 | 0.849 | -0.042 | 0.825 |
|  | Angular_R | 0.279 | 0.158 | -0.048 | 0.790 | 0.053 | 0.783 |
| SDS |  |  |  |  |  |  |  |
|  | STG_R | 0.324 | 0.099 | -0.024 | 0.895 | 0.228 | 0.225 |
|  | Pre/PostCG_L | 0.061 | 0.764 | -0.287 | 0.106 | -0.042 | 0.825 |
|  | mPFC | -0.368 | 0.059 | -0.077 | 0.671 | -0.095 | 0.618 |
|  | PCC/PCu | 0.153 | 0.445 | 0.181 | 0.314 | 0.051 | 0.789 |
|  | Angular_R | 0.240 | 0.229 | 0.065 | 0.720 | 0.007 | 0.971 |
| PCL_C |  |  |  |  |  |  |  |
|  | STG_R | 0.162 | 0.419 | 0.226 | 0.209 |  |  |
|  | Pre/PostCG_L | 0.283 | 0.153 | 0.032 | 0.860 |  |  |
|  | mPFC | ***-0.578*** | ***0.002***** | 0.030 | 0.868 |  |  |
|  | PCC/PCu | -0.146 | 0.467 | -0.029 | 0.871 |  |  |
|  | Angular_R | 0.164 | 0.414 | -0.134 | 0.458 |  |  |
| CAPS |  |  |  |  |  |  |  |
|  | STG_R | 0.091 | 0.653 |  |  |  |  |
|  | Pre/PostCG_L | 0.188 | 0.349 |  |  |  |  |
|  | mPFC | -0.037 | 0.854 |  |  |  |  |
|  | PCC/PCu | -0.105 | 0.602 |  |  |  |  |
|  | Angular_R | 0.285 | 0.150 |  |  |  |  |

** Significant spearman correlation at the group level (P < 0.01, Bonferroni corrected). R Right; L Left; STG superior temporal gyrus; Pre/PostCG pre- and post-central gyrus; mPFC medial prefrontal cortex; PCC/PCu Posterior cingulate cortex; PCu Precuneus
